# The reimbursement process in three national healthcare systems: variation in time to reimbursement of pembrolizumab for metastatic non-small cell lung cancer

**DOI:** 10.1186/s40545-023-00529-0

**Published:** 2023-02-16

**Authors:** Sarah Sharman Moser, Frank Tanser, Nava Siegelmann-Danieli, Lior Apter, Gabriel Chodick, Josie Solomon

**Affiliations:** 1 Maccabi Institute for Research and Innovation (Maccabitech), Maccabi Healthcare Services, 27 Hamered St, 6812509 Tel Aviv, Israel; 2 Lincoln International Institute of Rural Health, Lincoln Medical School, University of Lincoln, Brayford Way, Brayford Pool, Lincoln, LN6 7TS UK; 3 Sackler Faculty of Medicine, Tel Aviv University, Tel Aviv, Israel; 4 Department of Health Systems Management, Ben-Gurion University of the Negev, Beer-Sheva, Israel; 5 The School of Pharmacy, Joseph Banks Laboratories, University of Lincoln, Beevor Street, Lincoln, LN6 7DL UK

**Keywords:** Pembrolizumab, Reimbursement, Lung cancer, Health policy

## Abstract

In this article, we focus on the reimbursement process, and as an example, characterize the time to reimbursement of *pembrolizumab*, a PD-1 immune checkpoint inhibitor for treatment of metastatic NSCLC from publicly available websites, in three different healthcare systems: The National Institute for Health and Care Excellence (NICE) in the UK, the Pharmaceutical Benefits Advisory Committee (PBAC) in Australia, and the National Advisory Committee for the Basket of Health Services in Israel, all who have publicly funded health systems which include drug coverage. Our study found that there are substantial differences in time to reimbursement of pembrolizumab for the same conditions in different countries, with NICE and The National Advisory Committee for the Basket of Health Services in Israel approving one condition at the same time, Israel approving two conditions earlier than NICE, and PBAC lagging behind for every condition. These differences could be due to the differences in health policy systems and the many factors that affect reimbursement. Comparing the reimbursement process between different countries can highlight the challenges facing their health systems in early adoption of new treatments.

## Key points

- Our study found that there are substantial differences in time to reimbursement of pembrolizumab for the same conditions in different countries, with NICE and The National Advisory Committee for the Basket of Health Services in Israel approving one condition at the same time, Israel approving two conditions earlier than NICE, and PBAC lagging behind for every condition.
- These differences could be due to the differences in health policy systems and the many factors that affect reimbursement.
- Comparing the reimbursement process between different countries can highlight the challenges facing their health systems in early adoption of new treatments.

## Introduction

The high cost of new cancer therapies and limited budgets pose a unique challenge for resource allocation decisions across countries and healthcare systems [1]. In developed countries with national universal health insurance coverage, different methods have been established to assess new technologies in an orderly and transparent fashion before their inclusion into their list of services reimbursed using their allocated budget [2–4]. This process, known as the “fourth hurdle”, is an additional stage following registration of the technology in the country (and market authorization obtained) whereby a new technology has to demonstrate value for money and its effectiveness and safety need to be proven before being reimbursed from public funds. Many countries require further clinical evidence of efficacy beyond that provided by the manufacturer at market authorization, which may also consist of comparisons with similar drugs or accepted alternative therapies, and further clinical trials or post-marketing real-world data studies [5]. Many systems allow the inclusion of expensive treatments in the national health systems at no or minimum co-payment for the patient, allowing access to treatments that would otherwise be unobtainable for the vast majority of patients, either by creating separate health technology assessment pathways for cancer treatments or through exemptions policies which allow easier access to cancer treatments [6]. It is unknown whether time to reimbursement is similar or varies between countries. Several elements determine the incentives of payers to spend more funds on the adoption and diffusion of new medical technologies that need to be weighed up by decision-makers. Older drugs are generally safer due to their longer track record and the relative efficacy of newer drugs to older drugs may be marginal [7]. This manuscript argues for early adoption of anti-cancer treatments due to the limited availability of alternative treatments.

In this essay, we focus on a comparison of the reimbursement process, and as an example, characterize the time to reimbursement of *pembrolizumab*, a programmed death receptor-1 (PD-1) immune checkpoint inhibitor for treatment of metastatic non-small cell lung cancer (mNSCLC) from publicly available websites, in three different healthcare systems: The National Institute for Health and Care Excellence (NICE) in the United Kingdom (UK), the Pharmaceutical Benefits Advisory Committee (PBAC) in Australia, and the National Advisory Committee for the Basket of Health Services in Israel, who all have publicly funded health systems which include drug coverage. Pembrolizumab was chosen for this essay as the first-in-class immunotherapy drug used to treat mNSCLC in Israel. Comparing between different countries can highlight the challenges facing their health systems in early adoption of new treatments. Both NICE and PBAC base reimbursement decisions on cost-effectiveness evaluations and were chosen to be compared with Israel where a different system is used [8].

## Overview of the reimbursement process in three healthcare systems

All countries in this review have publicly funded health systems which include drug coverage, and have systems in place to assess new technologies (Table 1).

**Table 1:** Comparison of the reimbursement process for three healthcare services

	England	Australia	Israel
Who involved in decision-making	NICE—consisting of patient and health providers, academics, pharmaceutical and medical devices industries	PBAC—consisting of health care professionals, academics and consumer representatives	The National Advisory Committee for the Basket of Health Services—consisting of health care professionals, health ministry and health fund representatives, economists, hospital managers, lawyers, religious leaders and public representatives
Founded	1999	1960	1995
Threshold	Cost-effectiveness QALY £20,000–£30,000 (approximately US $24,000 to $36,000)	No information	No threshold
How long do appraisals take?	9–12 months	4–5 months	Committee meets once a year with a pre-determined budget, (the whole process take 3–4 months)
Other pathways or exemptions for cancer drugs	Exemptions, The Cancer Drugs Fund	No information	None
Recommendations made to	National Health Service	Australian Ministry of Health	Israel Ministry of Health
Time from application for first marketing approval to inclusion decision	23 months	No information	9 months
Out of pocket cost to oncology patients	Free	Free for patients receiving treatment in hospital. Co-payment for treatments purchased and received in the community	Free

The National Institute for Clinical Excellence is an independent organization set up by the British Government in England in 1999. In 2005 it joined with the Health Development Agency to become the National Institute for Health and Clinical Excellence, with the aim of deciding which drugs and treatments should be available on the National Health Service in England. NICE has acquired a high reputation internationally for evidence-based health technology assessments for the development of clinical guidelines and consists of employees from the National Health Services, patient and health providers, academics, pharmaceutical and medical devices industries. The main criterion for approval for NICE is cost-effectiveness [9], expressed as incremental cost per quality adjusted life-year (QALY) gained with thresholds set at £20,000–£30,000 (approximately US $24,000 to $36,000) for general models, however exceptions are made for cancer drugs that are generally not cost effective and many would not be approved for reimbursement according to this system [10, 11]. In 2011, the “Cancer Drugs Fund” was established to subsidize out-of-pocket expenses for drugs not recommended by NICE [6]; however, due to overspending it was restructured in 2016 and NICE took over responsibility for appraisals and funding to give stronger value for money and faster access to new effective treatments [12]. NICE reviews all oncology drugs and other technologies without a time limit for completing appraisals, and makes recommendations to the National Health Service for inclusion. Budget impact was not assessed by NICE until 2017 [13].

The PBAC in Australia consists of health care professionals, academics, health economists and consumer representatives and carries out independent health technology assessments considering which drugs should be listed in the national formulary and which vaccines included in the National Immunization Program [14]. Two sub-committees meet prior to PBAC meetings: the Economics Sub-committee which analyzes clinical and economic data, and the Drug Utilization Sub-committee which looks at budget impact data. Health technology assessments are based on cost-effectiveness, however no threshold has been published [15, 16]. There is no formal requirement for new technologies to be accessed and no time limit. These recommendations are then submitted to the Australian Ministry of Health and are usually accepted by the government although when the PBAC rejects a technology the government cannot then implement it. (The only exemption to this rule was when the Australian Government established the Herceptin program outside of the Pharmaceutical Benefits Scheme in order to fund trastuzumab for breast cancer in 2001 [17]). The PBAC has been chosen for this review as an example of another publicly funded healthcare system, and due to the fact that clinicians and manufacturers in Australia were concerned that the method used for drug selection and reimbursement may negatively affect patients’ access to new cancer drugs due to lower cost-effectiveness as compared to non-cancer treatments [18]. Although the point of using cost-effectiveness analysis is to ensure that drugs providing the highest cost-effectiveness are reimbursed, many patients would not be able to afford to pay the high prices of anti-cancer treatments privately. Guidelines are in place for a “rule of rescue” which allows the use of expensive products for severe diseases affecting few patients where no alternative treatments are available [19].

Israel has a system of universal healthcare as passed in the 1995 National Health Insurance Law which determines a standard list of healthcare services that all residents are entitled to receive via one of four national health funds (and hospitals) with a low co-payment system [20] (hence variation in prescribing due to “postcode” prescribing does not exist in Israel [21]). Israel has a unique method of centralized health technology assessment. Decisions on reimbursed technologies are made by a public committee (The National Advisory Committee for the Basket of Health Services) appointed by the Ministers of Health and Finance annually, to determine which new and innovative technologies and drugs will be recommended to the government to be included in the standard national formulary list and covered by the health funds from the following year onwards, according to a pre-allocated budget [22, 23]. Recommendations for incorporation of new technologies can be received from any interested party, including physicians, drug companies, patient-advocacy groups and uniquely, from individual citizens, and are submitted to the Ministry of Health from January to March of each year. Before The National Advisory Committee for the Basket of Health Services convenes, a portfolio of information is prepared for each technology including safety data, epidemiology data and costs, and in addition, each professional medical association (e.g., for oncology, hematology, cardiology, etc.) prioritizes the list of medications relevant to their specialization that have been submitted for inclusion in the national formulary that year, according to clinical advantage over present medications in the national formulary, irrespective of cost [24]. Budget impact is performed for all potential new treatments and the added value of each technology is compared to other technologies currently available in the national formulary. The National Advisory Committee for the Basket of Health Services which includes health care professionals, health ministry and health fund representatives, economists, hospital managers, lawyers, religious leaders and public representatives [25], makes a final evaluation and decision of which technologies will be incorporated, based on clinical values, social and ethical considerations between October and December of each year [24]. Committee members who have a conflict of interest do not take part in the decisions surrounding that particular technology. The committee then advises the Ministry of Health of which new technologies should be adopted into the national health insurance beginning in January of the following year [26]. The national list of reimbursed services is known as the national formulary. The pre-determined budget covers only a small fraction of the total amount of new technologies requested, in general after United States Food and Drug Administration (FDA) or European Medicines Agency (EMA) approval (The Health Ministry in Israel can register new medications after either FDA or EMA approval). For example, in 2020 the Committee had to decide how to allocate a budget of 500 million shekels (approximately US $140 million) for over 800 candidate technologies—mostly pharmaceuticals, and with a combined total cost of more than three billion shekels (approximately US $850 million)—resulting in 141 being added to the national formulary. Once an oncology treatment is incorporated into the national formulary for specific indications, all patients that meet the relevant criteria will receive it free of charge.

There may be differences in timing of health assessments between different countries corresponding to different times when the new technology may be added to health coverage. NICE and PBAC take approximately 9–12 months and 4–5 months, respectively, to carry out this process [27]. Length of time from application for first marketing approval to approval of inclusion decision was 23 months in England and 9 months in Israel [28].

## Reimbursement dates of pembrolizumab in three healthcare systems

There are currently four indications for use of pembrolizumab for mNSCLC (see Table 2). The first indication approved by the FDA in October 2015, was for second-line treatment of mNSCLC after progression of the disease with chemotherapy treatment, for patients with PD-L1 tumor proportion score (TPS) ≥ 1%, based on the Keynote-010 study [29]. It was then reimbursed in England in January 2017 [30] and in Israel in January 2018. The PBAC rejected reimbursement of this indication in Australia in 2016 [31].

**Table 2:** Dates of approval and reimbursement of pembrolizumab

Indication	FDA approval	NICE, England	PBAS, Australia	The National Advisory Committee for the Basket of Health Services, Israel
2L after disease progression with chemotherapy with PD-L1 TPS ≥ 1%	October 2015	January 2017	(Rejected in 2016)	January 2018
1L monotherapy for TPS ≥ 50%	October 2016	June 2017	July 2018 (with PS 0–1)	January 2017
1L in combination with chemotherapy—non-squamous cell carcinoma	August 2018	January 2019	July 2019 (with PS 0–1)	January 2019
1L in combination with chemotherapy—squamous cell carcinoma	October 2018	September 2019	–	January 2019

*FDA* Food and Drug Administration, *NICE* The National Institute for Health and Care Excellence, *PBAC* Pharmaceutical Benefits Advisory Committee, *1L* first line, *2L* second line, *PD-L1* programmed death-ligand 1, *TPS* tumor proportion score, *PS* performance score

Approval for the use of pembrolizumab as second-line treatment was then followed by approval as first-line monotherapy for patients with a TPS ≥ 50% and no actionable genomic driver mutations (i.e., EGFR, ALK or ROS1), and was given in October 2016 by the FDA, based on the Keynote-024 study [32]. In Israel this indication was approved soon after in January 2017, in England in June 2017 [33, 34] and in Australia in July 2018 (reimbursement started from November and only for patients with performance score 0–1) [35].

Pembrolizumab in combination with chemotherapy for first-line treatment for non-squamous and squamous NSCLC (based on Keynote-189 and Keynote 407) was first approved by the FDA in August [36] and October 2018, respectively [37]. Pembrolizumab was approved in Israel for this indication for both histology types in January 2019, in England for non-squamous in January 2019 [38, 39] and for squamous in September 2019 [40]. The PBAC approved pembrolizumab for first-line combination therapy with chemotherapy for patients with non-squamous cell disease for only those with a performance score of 0–1 in July 2019 [41] and as of writing has not approved first-line combination therapy for squamous cell carcinoma.

## Discussion

The rate-limiting step for reimbursement of new drugs is government policies, whether medications are admitted to the national formulary or not, and their success in penetrating the market will be dependent on many reasons including prescriber characteristics, familiarity of the new treatments, the presence of alternative therapies, evidence of improved clinical outcomes and marketing efforts of pharmaceutical companies [42]. Limitations to prescribing include the requirement of prior authorization, specialist use only in secondary care or as a second-line therapy after failure of a (usually) cheaper drug [43].

Nonetheless, our review found that there are substantial differences in time to reimbursement of pembrolizumab for the same indications in different countries, with NICE and The National Advisory Committee for the Basket of Health Services in Israel approving one condition at the same time, Israel approving two conditions earlier than NICE, and PBAC lagging behind for every condition. A previous study reported that NICE recommended 87.4% of drug submissions as compared with 54.3% from PBAC [44]. These differences could be due to the differences in health policy systems and the many factors that affect reimbursement. One paper reported that countries with social health insurance systems tended to reimburse drugs more than other countries, all countries were more likely to make a favorable decision if NICE had given approval previously, and in addition, cost-effective drugs were more likely to be approved for use [45]. Prior to 2005, the UK had been accused of slower uptake of new medicines than other OECD countries [46–48]. This coupled with “postcode prescribing” led to a change in how medication was reimbursed in England [21]. One purpose of NICE was that all new technologies approved are prescribable in all areas and not dependent on formulary inclusion by the therapeutics board of a health authority. In addition, the Cancer Fund in the UK was established in order to help patients gain faster access to cancer drugs [12].

Apart from second-line treatment, Israel approved reimbursement in the health system for pembrolizumab for first-line monotherapy and in combination with chemotherapy for squamous cell lung cancer before NICE in England and PBAC in Australia. An important note: the dates of reimbursement reported here are when the committees published their proposals and not date of implementation. In Israel the implementation is immediate whereas in England and Australia it can take several months [49], emphasizing that Israel reimbursed these indications even earlier than the other countries.

Unlike other countries, part of the ability of Israel to be an early adopter of new treatments is the flexibility of the National Advisory Committee for the Basket of Health Services, whereby the committee meets annually to recommend new technologies to be included into the national formulary, influenced by treatment effectiveness, lack of therapeutic alternatives, cost and others, also including difficult ethical considerations such as including a cheaper technology or screening for many with the greatest impact on the population, or an expensive oncology treatment to treat just a few, but with lower budget impact [22, 50]. By March of each year, new technologies are submitted to the Ministry of Health, in the following months data are collected including safety data, data concerning added benefit, drug preference and budget impact, and in October to December the committee convenes to debate which technologies will be added to the national formulary with a decision made by January of the following year. This deadline ensures a timely addition to the national formulary each year and a relatively short time from submission to acceptance. All new technologies for a particular year are considered simultaneously and competitively for funding using this ranking system, subject to the budget constraint. This method of budget impact as compared to cost-effectiveness allows flexibility in choosing technologies to be included, sensitive to changes in the health of the nation. This usually leads to a balance in the national formulary, including a variety of technologies. This is considered unique by many health care analysts in contrast to other systems like those is the UK and Australia that perform ad hoc health technology assessments and determine whether to include a new technology due to cost implications (i.e., limited by QALY), irrespective of other new treatments, and where funding decisions may be blocked to restrain costs [51]. In addition, in Israel, anyone can make a request for a new technology to be assessed, including drug companies, physicians and patient groups, in contrast to the PBAC that only allows pharmaceutical companies to request assessment. Another advantage of the Israeli system might be that the budget for the following year is guaranteed, so there is no need to request additions to the health budget. An additional factor in the early adoption of pembrolizumab in Israel could be due to the orientation of the National Advisory Committee for the Basket of Health Services towards adoption of oncology and hematology drugs (approximately 40–50% of the annual budget) [52]. A disadvantage of this system which only uses budget impact analysis, is that it does not use an objective measure like cost-effective analysis, and due to budget limitations, cancer drugs may be reimbursed at the expense of other cheaper and necessary drugs in the health system which could have the potential to treat many more people. A further analysis could determine whether Israel is also an early-adopter of non-oncology drugs.

Israel has long been a country eager to adopt new pharmaceutical and electronic technologies in the health system [53]. With a relatively advanced computerized healthcare system, all the health funds use electronic medical records and store the information in electronic databases, allowing accurate prediction of potential numbers of patients eligible to receive new technologies for a particular disease, and hence providing accurate information for budget impact calculations.

As mentioned above, most countries base their decisions surrounding whether to reimburse new technologies on cost-effectiveness, however it has been recognized that there are many other issues and values necessary to include in these decisions [54]. Many healthcare systems choose the “health maximization principle” that allows the maximum health for the maximum number of people in a population, however this may prevent those worse off receiving treatments (i.e., for cancer patients) who may not be able to privately fund their treatment. Principles such as need, clinical effectiveness, transparency and appeal mechanisms are some of the issues necessary for a fair system that have been implemented in many health systems, however other values such as social values, moral and ethical principles are harder to define and implement, and religious values heavily influence decisions in Israel [55]. For example, a law passed in Parliament in Israel in 1996 mandates that bystanders *must* give assistance to people in danger as much as they are able, demonstrating the high value placed on human life, and this is also reflected by the high proportion of technologies approved in the annual formulary for cancer treatments, that have potential life-saving or life-prolongation value. NICE published a document called the “Social Value Judgements” that includes principles for the development of NICE guidance such as moral principles (including respect for autonomy, non-maleficence and beneficence) and distributive justice (using a utilitarian and egalitarian approach) [56].

A systemic review identified social values proposed or used in making resource allocation decisions for new health technologies, however most of the studies included looked at each value separately without prioritizing them against each other [54]. The severity of illness principle proposes allocating resources to those “worse off” as long as they could realize a large health gain through treatment and those in immediate need would include those with a life-threatening or terminal illness. Age is another factor with some studies recommending alllocating resources to younger patients in order to allow them a normal life-span. Opinions varied regarding whether resources should be allocated to patients who were responsible for their disease due to unhealthy behavior (e.g., lung cancer patients who smoked) or only to those that their illness was caused naturally. Other values included treating those that had dependents as opposed to those without, time already waited for treatment and health gain (amount of health gain and expected outcome) expected for treatment. The review agreed that none of the following should be taken into account in decision-making: socioeconomic status, sex, marital status or religion. All these values carry significant resource implications were considered in isolation, limiting the ability to make decisions based on their integration.

A recent systemic review analyzed qualitatively 40 studies to determine the appropriate criteria used in health technologies priority-setting models in the world [57]. The authors reported that they found many different criteria for selecting health technologies including health outcomes, technology alternatives, economic aspects and target populations. They concluded that there was a need for a multi-criteria approach for decision-making including new technologies in healthcare services, but did not report an integrated system that takes into account all the different aspects.

While all these social and ethical principles are important, their relative importance differs between countries and no guidance of their relative weighting exists. In Israel, the members of the National Advisory Committee for the Basket of Health Services are expected to take into account conflicting ethical and social principles within the decision-making process, however no scoring system exists to quantitate these principles.

A study in 2012 proposed integrating these values in a model “Value for Money Chart” to give a final score, which would allow for transparency and further support the decision-making process [58]. The model proposed incorporating the following principles within a points system: lives saved, life-prolongation benefits, quality of life gains, social benefits (for example targeted to minorities or reducing health gaps) and if this technology could not to be funded would the patients be able to pay for it themselves. This system involved rating each technology on each value per year and then summing the points to get a final score, allowing prioritization of different technologies against each other. The system is obviously very complex, with many questions that could be asked concerning how to grade each section, what level of evidence each is based on and with some of the sections open to interpretation, however the points system could reflect the committee’s subjective priorities. It is important to note, these models would not be in place of the various committees, but could support decision-makers in these difficult decisions and allow transparency with stake holders and the public. This model could not only help prioritize technologies against each other, but also help assess individual technologies.

### Study limitations

In this paper, we assessed three countries’ health policies for reimbursement of pembrolizumab, all with social healthcare systems; therefore, our conclusions may not be generalizable for other drugs or other types of healthcare systems. Significant inter-country variability exists in health technology assessment recommendations [59–63] which may be due to different data sets submitted to different agencies, the inclusion or exclusion of different clinical trials and direct or indirect data [60] and also interpretation of the evidence provided and how to deal with uncertainty [64]. This manuscript argues for early adoption of anti-cancer treatments due to the limited availability of alternative treatments, but a further topic of importance but out of the scope of this essay could discuss the ethics in approval of very expensive anti-cancer drugs which may have significant progression free survival but non-significant overall survival, and which would impact the ability to include other cheaper technologies to treat many people due to budget limitations.

## Conclusion

Our comparative analysis of time to reimbursement of pembrolizumab has revealed the challenges faced by three healthcare systems in adoption of new treatments. The most important challenge was budget limitations among many factors that are taken into account in the reimbursement process, and length of time from registration to reimbursement in the public health system of each country.

It is unclear whether it is possible to encourage earlier access to necessary and life-saving cancer treatments in countries where the reimbursement process is longer, as each country has the right to accept or reject treatments according to its own criteria and needs, but certainly this subject needs to be debated.

According to the Israeli National Health Insurance act, the government sets a capped annual budget for new health technologies while the prioritization of the new technologies is set by an independent committee once a year according to various clinical, ethical, and social considerations. While this system is less objective than cost-effectiveness analysis, it allows for a relatively rapid response to changing healthcare needs [65].

## Data Availability

Not applicable.

## Abbreviations

ALK: Anaplastic lymphoma kinase
EGFR: Epidermal growth factor receptor
EMA: European Medicines Agency
FDA: United States Food and Drug Administration
mNSCLC: Metastatic non-small cell lung cancer
NICE: National Institute for Health and Care Excellence
PBAC: Pharmaceutical Benefits Advisory Committee
PD-1: Programmed death-1
PS: Performance score
QALY: Quality adjusted life-year
ROS1: ROS protooncogene-1 receptor tyrosine kinase
TPS: Tumor proportion score
UK: United Kingdom

## References

[1] Leopold C (2014). Pharmaceutical policy analysis—a European perspective on pricing and reimbursement in challenging times.

[2] Fischer KE (2012). A systematic review of coverage decision-making on health technologies—evidence from the real world. Health Policy.

[3] Panteli D, Eckhardt H, Nolting A, Busse R, Kulig M (2015). From market access to patient access: overview of evidence-based approaches for the reimbursement and pricing of pharmaceuticals in 36 European countries. Health Res Policy Syst.

[4] Allen N, Liberti L, Walker SR, Salek S (2017). A comparison of reimbursement recommendations by European HTA agencies: is there opportunity for further alignment?. Front Pharmacol.

[5] Barnieh L, Manns B, Harris A, Blom M, Donaldson C, Klarenbach S (2014). A synthesis of drug reimbursement decision-making processes in organisation for economic co-operation and development countries. Value Health.

[6] Neumann PJ, Bliss SK, Chambers JD (2012). Therapies for advanced cancers pose a special challenge for health technology assessment organizations in many countries. Health Aff.

[7] Alexander GC, O'Connor AB, Stafford RS (2011). Enhancing prescription drug innovation and adoption. Ann Intern Med.

[8] Wang S, Gum D, Merlin T (2018). Comparing the ICERs in medicine reimbursement submissions to NICE and PBAC—does the presence of an explicit threshold affect the ICER proposed?. Value Health.

[9] Dakin H, Devlin N, Feng Y, Rice N, O'Neill P, Parkin D (2015). The influence of cost-effectiveness and other factors on nice decisions. Health Econ.

[10] Buxton MJ (2006). Economic evaluation and decision making in the UK. Pharmacoeconomics.

[11] Ewbank L, Omojomolo D, Sullivan K, McKenna H. The rising cost of medicines to the NHS. The Kings Fund. 2018:2018-04.

[12] NHS. Appraisal and funding of cancer drugs from July 2016 (including the new cancer drugs fund); 2016. https://www.england.nhs.uk/wp-content/uploads/2013/04/cdf-sop.pdf.

[13] NICE. Budget impact test; 2017. https://www.nice.org.uk/about/what-we-do/our-programmes/nice-guidance/nice-technology-appraisal-guidance/budget-impact-test.

[14] Parkinson BT. Pharmaceutical policy in Australia: developing methods to manage uncertainty in health technology assessment; 2015.

[15] Levy AR, Mitton C, Johnston KM, Harrigan B, Briggs AH (2010). International comparison of comparative effectiveness research in five jurisdictions. Pharmacoeconomics.

[16] George B, Harris A, Mitchell A (2001). Cost-effectiveness analysis and the consistency of decision making. Pharmacoeconomics.

[17] PBS. Trastuzumab, powder for I.V. infusion, 150 mg, Herceptin®, November 2008. https://www.pbs.gov.au/info/industry/listing/elements/pbac-meetings/psd/2008-11/pbac-psd-trastuzumab-nov08.

[18] Chim L, Kelly PJ, Salkeld G, Stockler MR (2010). Are cancer drugs less likely to be recommended for listing by the Pharmaceutical Benefits Advisory Committee in Australia?. Pharmacoeconomics.

[19] PBAC. Guidelines for preparing submissions to the Pharmaceutical Benefits Advisory Committee; 2015. https://pbac.pbs.gov.au/content/information/archived-versions/pbac-guidelines-v4-5.pdf.

[20] Chinitz D, Israeli A (1997). Health reform and rationing in Israel. Health Aff.

[21] Parsons A, Johnstone A (2001). Postcode prescribing and the Human Rights Act 1998. J R Soc Med.

[22] Sax P (2014). The shaping of pharmaceutical governance: the Israeli case. Israel J Health Policy Res.

[23] Shalev C, Chinitz D (2005). Joe public v. the general public: the role of the courts in Israeli health care policy. J Law Med Ethics.

[24] Wolf I, Waissengrin B, Zer A, Bernstein-Molho R, Rouvinov K, Cohen JE (2022). Implementation of the ESMO-magnitude of clinical benefit scale: real world example from the 2022 Israeli National Reimbursement Process. ESMO Open..

[25] Clarfield AM, Manor O, Nun GB, Shvarts S, Azzam ZS, Afek A (2017). Health and health care in Israel: an introduction. Lancet.

[26] Shani S, Siebzehner MI, Luxenburg O, Shemer J (2000). Setting priorities for the adoption of health technologies on a national level—the Israeli experience. Health Policy.

[27] Fischer KE, Heisser T, Stargardt T (2016). Health benefit assessment of pharmaceuticals: an international comparison of decisions from Germany, England, Scotland and Australia. Health Policy.

[28] Chapman S, Paris V, Lopert R. Challenges in access to oncology medicines: policies and practices across the OECD and the EU. 2020.

[29] FDA. FDA approves pembrolizumab for patients with non-small cell lung cancer; 2015. https://www.cancer.gov/news-events/cancer-currents-blog/2015/pembrolizumab-nsclc#:~:text=On%20October%202%2C%20the%20Food,progressed%20after%20platinum%2Dbased%20chemotherapy.

[30] NICE. Pembrolizumab for treating PD-L1-positive non-small-cell lung cancer after chemotherapy TA428; 2017. https://www.nice.org.uk/guidance/ta428/chapter/1-Recommendations.

[31] PBAC. Pembrolizumab (NSCLC): powder for injection 50mg; Keytruda; 2016. https://www.pbs.gov.au/industry/listing/elements/pbac-meetings/psd/2016-11/files/pembrolizumab-nsclc-psd-november-2016.pdf.

[32] FDA. Pembrolizumab (KEYTRUDA) checkpoint inhibitor; 2016. https://www.fda.gov/drugs/resources-information-approved-drugs/pembrolizumab-keytruda-checkpoint-inhibitor.

[33] NICE. Pembrolizumab for untreated PD-L1-positive metastatic non-small-cell lung cancer TA447; 2017. https://www.nice.org.uk/guidance/ta447. 41406309

[34] NICE. Pembrolizumab for untreated PD-L1-positive metastatic non-small-cell lung cancer TA531; 2018 [9th December 2021]. https://www.nice.org.uk/guidance/ta531/chapter/1-Recommendation. 41406309

[35] PBAC. Pembrolizumab (NSCLC): powder for injection 50 mg; solution concentrate for I.V. infusion 100 mg in 4 mL; Keytruda; 2018. https://www.pbs.gov.au/industry/listing/elements/pbac-meetings/psd/2018-07/files/pembrolizumab-nsclc-psd-july-2018.pdf.

[36] FDA. FDA grants regular approval for pembrolizumab in combination with chemotherapy for first-line treatment of metastatic nonsquamous NSCLC; 2018. https://www.fda.gov/drugs/resources-information-approved-drugs/fda-grants-regular-approval-pembrolizumab-combination-chemotherapy-first-line-treatment-metastatic.

[37] FDA. FDA approves pembrolizumab in combination with chemotherapy for first-line treatment of metastatic squamous NSCLC; 2018. https://www.fda.gov/drugs/fda-approves-pembrolizumab-combination-chemotherapy-first-line-treatment-metastatic-squamous-nsclc.

[38] NICE. Pembrolizumab with pemetrexed and platinum chemotherapy for untreated, metastatic, non-squamous non-small-cell lung cancer TA557; 2019. https://www.nice.org.uk/guidance/ta557. 40737472

[39] NICE. Pembrolizumab with pemetrexed and platinum chemotherapy for untreated, metastatic, non-squamous non-small-cell lung cancer TA683; 2021. https://www.nice.org.uk/guidance/ta683/chapter/1-Recommendations. 40737472

[40] NICE. Pembrolizumab with carboplatin and paclitaxel for untreated metastatic squamous non-small-cell lung cancer TA600; 2019. https://www.nice.org.uk/guidance/ta600. 40455901

[41] PBAC. Pembrolizumab solution concentrate for I.V. infusion 100 mg in 4 mL; Keytruda. 2019. https://www.pbs.gov.au/industry/listing/elements/pbac-meetings/psd/2019-07/files/pembrolizumab-nsclc-psd-july-2019.pdf.

[42] Lublóy Á (2014). Factors affecting the uptake of new medicines: a systematic literature review. BMC Health Serv Res.

[43] Medlinskiene K, Tomlinson J, Marques I, Richardson S, Stirling K, Petty D (2021). Barriers and facilitators to the uptake of new medicines into clinical practice: a systematic review. BMC Health Serv Res.

[44] Clement FM, Harris A, Li JJ, Yong K, Lee KM, Manns BJ (2009). Using effectiveness and cost-effectiveness to make drug coverage decisions: a comparison of Britain, Australia, and Canada. JAMA.

[45] Pujolras LM, Cairns J (2015). Why do some countries approve a cancer drug and others don’t?. J Cancer Policy.

[46] Pharmaceutical industry competitiveness task force; 2001. https://www.rieti.go.jp/jp/events/bbl/data/pictf.pdf.

[47] Sciences OfL. Life sciences competitiveness indicators. 2019. https://assets.publishing.service.gov.uk/government/uploads/system/uploads/attachment_data/file/811347/life-sciences-competitiveness-data-2019.pdf.

[48] Chauhan D, Mason A (2008). Factors affecting the uptake of new medicines in secondary care—a literature review. J Clin Pharm Ther.

[49] Fund TK. Access to new medicines in the English NHS; 2020. https://www.kingsfund.org.uk/publications/access-new-medicines-english-nhs.

[50] Guttman N, Shalev C, Kaplan G, Abulafia A, Bin-Nun G, Goffer R (2008). What should be given a priority–costly medications for relatively few people or inexpensive ones for many? The Health Parliament public consultation initiative in Israel. Health Expect.

[51] Shaw B. Deferring PBAC decisions: industry view. An independent review. 2012:3.

[52] News IH. Cancer patients fear ahead of drug basket discussions: “who will make our voices heard in committee?” 2021. https://www.israelhayom.co.il/health/article/4850070.

[53] Beyar R, Zeevi B, Rechavi G (2017). Israel: a start-up life science nation. Lancet.

[54] Stafinski T, Menon D, Marshall D, Caulfield T (2011). Societal values in the allocation of healthcare resources. Patient Patient-Cent Outcomes Res.

[55] Jotkowitz AB, Agbaria R, Glick SM (2017). Medical ethics in Israel—bridging religious and secular values. Lancet.

[56] NICE. The principles that guide the development of NICE guidance and standards. https://www.nice.org.uk/about/who-we-are/our-principles.

[57] Mobinizadeh M, Raeissi P, Nasiripour AA, Olyaeemanesh A, Tabibi SJ (2016). The health systems’ priority setting criteria for selecting health technologies: a systematic review of the current evidence. Med J Islam Repub Iran.

[58] Golan O, Hansen P (2012). Which health technologies should be funded? A prioritization framework based explicitly on value for money. Israel J Health Policy Res.

[59] Nicod E, Kanavos P (2012). Commonalities and differences in HTA outcomes: a comparative analysis of five countries and implications for coverage decisions. Health Policy.

[60] Spinner DS, Birt J, Walter JW, Bowman L, Mauskopf J, Drummond MF (2013). Do different clinical evidence bases lead to discordant health-technology assessment decisions? An in-depth case series across three jurisdictions. CEOR.

[61] Lim CS, Lee Y-G, Koh Y, Heo DS (2014). International comparison of the factors influencing reimbursement of targeted anti-cancer drugs. BMC Health Serv Res.

[62] Salas-Vega S, Bertling A, Mossialos E (2016). A comparative study of drug listing recommendations and the decision-making process in Australia, the Netherlands, Sweden, and the UK. Health Policy.

[63] Allen N, Walker SR, Liberti L, Salek S (2017). Health technology assessment (HTA) case studies: factors influencing divergent HTA reimbursement recommendations in Australia, Canada, England, and Scotland. Value Health.

[64] Nicod E (2017). Why do health technology assessment coverage recommendations for the same drugs differ across settings? Applying a mixed methods framework to systematically compare orphan drug decisions in four European countries. Eur J Health Econ.

[65] Rosen B, Waitzberg R, Israeli A (2021). Israel’s rapid rollout of vaccinations for COVID-19. Israel J Health Policy Res.

